# Effect of facemask oxygenation with and without positive pressure ventilation on gastric volume during anesthesia induction in patients undergoing laparoscopic cholecystectomy or partial hepatectomy: a randomized controlled trial

**DOI:** 10.1186/s12871-022-01958-1

**Published:** 2022-12-30

**Authors:** Guangting He, Liyun Ma, Ke Tian, Yuqi Cao, Zaisheng Qin

**Affiliations:** Department of Anesthesiology, NanFang Hospital, Southern Medical University, 1838 Guangzhou Avenue North, Guangzhou, 510515 People’s Republic of China

**Keywords:** Facemask oxygenation, Gastric volume, Anesthesia induction, Laparoscopic surgery

## Abstract

**Background:**

Studies focusing on the relationship between gastric volume and facemask oxygenation without ventilation during apnea in anesthesia induction are scarce. This study compared the change in gastric volume during apnea in anesthesia induction using facemask ventilation and facemask oxygenation without ventilation in adults undergoing laparoscopic surgery.

**Methods:**

In this prospective, randomized, double-blinded trial, 70 adults undergoing laparoscopic surgery under general anesthesia were divided into two groups to receive facemask oxygenation with and without ventilation for 60 seconds after loss of consciousness. Before anesthesia induction and after endotracheal intubation, the gastric antral cross-sectional area was measured with ultrasound imaging. Arterial blood gases were tested at baseline (T1), after preoxygenation (T2), after loss of consciousness (T3), and before and after endotracheal intubation (T4 and T5, respectively).

**Results:**

Sixty patients were included (ventilation *n* = 30; non ventilation *n* = 30, 10 patients were excluded). The median [IQR] change of gastric antral cross-sectional area in ventilation group was significantly higher than in non ventilation group (0.83 [0.20 to 1.54] vs. 0.10 [− 0.11 to 0.56] cm_2_, *P* = 0.001). At T4 and T5, the *P*aO_2_ in ventilation group was significantly higher than in non ventilation group (T4: 391.83 ± 61.53 vs. 336.23 ± 74.99 mmHg, *P* < 0.01; T5: 364.00 ± 58.65 vs. 297.13 ± 86.95 mmHg, *P* < 0.01), while the *P*aCO_2_ in non ventilation group was significantly higher (T4: 46.57 ± 5.78 vs. 37.27 ± 6.10 mmHg, *P* < 0.01; T5: 48.77 ± 6.59 vs. 42.63 ± 6.03 mmHg, *P* < 0.01) and the pH value in non ventilation group was significantly lower (T4: 7.35 ± 0.029 vs 7.42 ± 0.047, *P* < 0.01; T5: 7.34 ± 0.033 vs 7.39 ± 0.044, *P* < 0.01). At T4, the HCO_3_^−^ in non ventilation group was significantly higher (25.79 ± 2.36 vs. 23.98 ± 2.18 mmol l^− 1^, *P* < 0.01).

**Conclusions:**

During apnoea, the increase in gastric volume was milder in patients undergoing facemask oxygenation without ventilation than with positive pressure ventilation.

**Trial registration:**

ChiCTR2100054193, 10/12/2021, Title: “Effect of positive pressure and non-positive pressure ventilation on gastric volume during induction of general anesthesia in laparoscopic surgery: a randomized controlled trial”. Website: https://www.chictr.ogr.cn.

## Introduction

The clinical application of laparoscopic surgery is becoming increasingly popular, and general anesthesia with endotracheal intubation is a universal and ideal anesthetic modality [1]. Facemask ventilation is a routine procedure for maintaining adequate oxygenation during apnea in general anesthesia induction. However, in patients undergoing laparoscopic surgery, the consequent change in gastric volume is a common concern of anesthesiologists and surgeons. Gastric insufflation may affect the surgical visual field, prolong the operation time and also cause gastric contents regurgitation, aspiration pneumonitis and stomach perforation, thereby increasing the mortality risk [2–4].

A high inspiratory pressure causes gastric insufflation during facemask ventilation. Facemask preoxygenation and oxygenation without ventilation, that is apneic oxygenation, are typically used in rapid-sequence induction to prevent gastric content regurgitation and pulmonary aspiration. Apneic oxygenation is the passive delivery of oxygen into the alveoli through an open airway during apnea. In an apneic patient, the oxygen in the alveoli diffuses into the pulmonary blood stream, bringing the alveoli to a state of subatmospheric pressure [5]. This pressure gradient allows the oxygen supplied to the upper airway via a nasal duct or facemask to flow to the alveoli.

During anesthesia induction, different ventilation modalities can result in different degrees of gastric insufflation [6, 7]. However, studies focusing on the relationship between gastric volume and facemask oxygenation without ventilation during apnea in anesthesia induction in patients undergoing laparoscopic surgery are still scarce.

In previous studies, the occurrence of gastric insufflation was determined by auscultation with a stethoscope over the epigastrium; however, the reliability and accuracy of this method are uncertain [8, 9]. With abdominal ultrasound, we can detect the excessive presence of air in the stomach and accurately estimate the gastric volume through the measurement the gastric antral cross sectional area (CSA) [10–13].

In this prospective study, we aimed to compare the effects on gastric volume of facemask ventilation and facemask oxygenation without ventilation during apnea in anesthesia induction, in adults scheduled for laparoscopic cholecystectomy or partial hepatectomy.

## Methods

### Ethics

The study protocol was registered at the Chinese Clinical Trial Registry (ChiCTR2100054193) after approval by the Medical Ethics Committee of NanFang Hospital of the Southern Medical University, Guangzhou, China (NFEC-2021-401; chairperson, Yu Zhang; date of approval: December 2, 2021). All participants included in the study signed their informed consents.

### Study design and participants

This study is a single-center, prospective, randomized, double-blinded, controlled trial of patients scheduled for elective laparoscopic surgery under general anesthesia, from December 2021 to May 2022, in the Department of Anesthesiology, NanFang Hospital, Southern Medical University.

The inclusion criteria were as follows: physical status I-II following the American Society of Anesthesiologists (ASA) classification, age between 18 and 65 years, Mallampati grade I-III, BMI < 30 kg m^− 2^, adequate fasting status (8 h for solids and 2 h for clear liquids), and undergoing elective laparoscopic surgery. Drugs affecting the gastrointestinal dynamics, including trimebutine maleate, metoclopramide, domperidone, sisaprib, mosaprib, atropine, anisodamine, alossiron, ondansiron, grassiron and pinaverium bromide, were suspended the day before surgery. The exclusion criteria were as follows: allergy to the drugs involved in the study, known or predicted difficulty of intubation, risk of upper respiratory tract obstruction, cardiopulmonary dysfunction, cerebrovascular disease, gastroesophageal regurgitation, history of esophageal or gastrointestinal trauma or surgery, diabetes mellitus, and pregnancy. Withdrawal criteria included patient’s choice, change of surgical plan to open surgery, failure to obtain clear ultrasonographic images, facemask ventilation difficulty, more than one endotracheal intubation, SpO_2_ < 90%, and occurrence of gastric content regurgitation or aspiration.Patients were identified according to the inclusion criteria in the ward the day before surgery. The eligible patients were fully aware of the study protocol and signed written informed consent forms after acquiring the consent of the participant.

### Randomization and masking

A computer-generated random number table in a 1:1 ratio created by an independent investigator was used for randomization and allocation. The allocation was numbered in sequence and sealed in light-tight envelopes by the corresponding author; the attending anesthesiologist unfolded the envelopes 10 min before the patient entered the operating room. The patients were randomly allocated into two groups: positive and non-positive pressure facemask ventilation (ventilation group and non ventilation group, respectively).The patients, surgeons, and sonographer were blinded to patient group allocation. The attending anesthesiologist was not blinded; however, he was not aware of the study aim and did not participated in the gastric ultrasonography or data analysis.

### Procedures

In the operation room, intravenous access to an upper limb was established. Standard monitoring including electrocardiography, oxygen saturation (SpO_2_) measurement, non-invasive blood pressure monitoring were performed. Arterial puncture catheterization under local anesthesia was conducted to monitor invasive blood pressure. All patients were preoxygenated with pure oxygen for 3 min at the beginning of general anesthesia induction, at a flow rate of 8 L/min. Propofol and remifentanil were intravenously administered for anesthesia induction in the target-controlled infusion mode. After loss of consciousness and intravenous injection of rocuronium (1.0 mg kg^− 1^), a two-handed jaw-thrust technique was used to open the airway. Ventilation group underwent a pressure-controlled ventilation mode through a facemask, and the inspiratory pressure was set to 20 cmH_2_O at a rate of 15 breaths min^− 1^.The patients in non ventilation group were oxygenated via s facemask at an oxygen flow rate of 8 L/min, without any other positive pressure. Sixty seconds later, the trachea was intubated for mechanical ventilation. Atropine was administered when the heart rate was < 45 beats min^− 1^. Patients with hypotension (systolic pressure < 80 mmHg or mean arterial pressure < 60 mmHg) were treated with ephedrine. Propofol and remifentanil were continued to use in anesthesia maintenance.

**Fig. 1:**
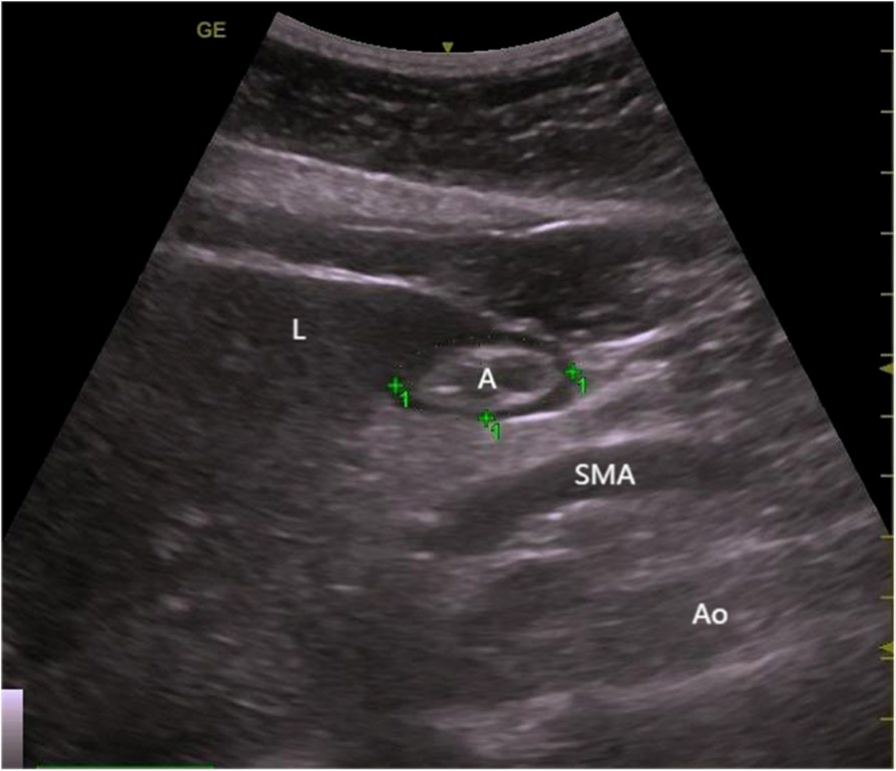
Ultrasonographic image of the gastric antrum. A: gastric antrum: L: liver; SMA: superior mesenteric artery; Ao: abdominal aorta

Before the anesthesia induction, the gastric ultrasonography was conducted with patients placed in the supine position. A portable color-Doppler ultrasound system (GE Healthcare, Chicago, IL, USA) with a 2-5-MHz ultrasonic transducer under the xiphoid process was used to scan the sagittal plane of gastric antrum. The sonographer adjusted the image to contain the gastric antrum, along the edge of left liver lobe, anterior to sagittal section of the abdominal aorta and superior mesenteric artery (Fig. 1). The anteroposterior and craniocaudal diameters of the antrum (D1 and D2, respectively) were measured from three images taken between contraction, to obtain a mean value. We used the following formula to calculate the gastric antral CSA:

$$\textrm{CSA}=\frac{\pi \times \textrm{D}1\times \textrm{D}2}{4}$$

After endotracheal intubation, the same sonographer, blinded to the allocation of the patient, repeated the measurement of the gastric antrum CSA at the same superficial location.

Arterial blood gas analysis was performed and recorded by an investigator who was blind to the group allocation at baseline (T1), after preoxygenation (T2), at loss of consciousness (T3), before endotracheal intubation (T4), and after endotracheal intubation (T5).

### Outcomes

The primary outcome was the change in the gastric antral CSA. The secondary outcomes included gastric antral CSA after induction, incidence of gastric insufflation defined by the same surgeon, arterial partial pressure of oxygen (*P*aO_2_), partial pressure of carbon dioxide (*P*aCO_2_), the pH value and bicarbonate (HCO_3_^−^) at T1, T2, T3, T4, and T5.

### Statistical analysis

The intervention and control groups were the non ventilation group and ventilation group, respectively. The change in gastric antral CSA was the main outcome; according to our pre-trial findings of 10 patients per group, the increase in gastric antral CSA during induction was 0.012 ± 0.571 cm^2^ and 0.766 ± 0.785 cm^2^ without and with positive pressure, respectively. The procedures of pre-trail was the same as this study and was conducted after obtaining ethical clearance. With a type I error α (two-tailed) = 0.05 and test efficiency 1-β = 0.9, the sample size was estimated with the formula of the two-population mean hypothesis test designed by PASS 15.0. The sample size required was determined to be 18 patients per group; assuming a withdrawal rate of 10%, 23 patients were suggested in each group.

The categorical data are presented as numbers or percentages (*n* [%]) and analyzed using the chi-square test for comparisons between groups. The continuous data are presented as mean ± SD or median [IQR]), as appropriate. The normal distribution of the data was evaluated using the Shapiro-Wilk test.

For the indicators that followed a normal distribution and had uniform variance in both groups, the independent sample t-test was used for the analysis; otherwise, the Mann-Whitney U test. Two-way repeated measures ANOVA with the Bonferroni post hoc-test and correction for *P* value was used to compare the data at multiple time points between groups. We used Statistical Package for Social Sciences (SPSS) version 26.0 (IBM Corp, Armonk, NY, USA) for statistical analyses and consider all *P* values statistically significant at the two-sided < 0.05 level.

## Results

**Fig. 2:**
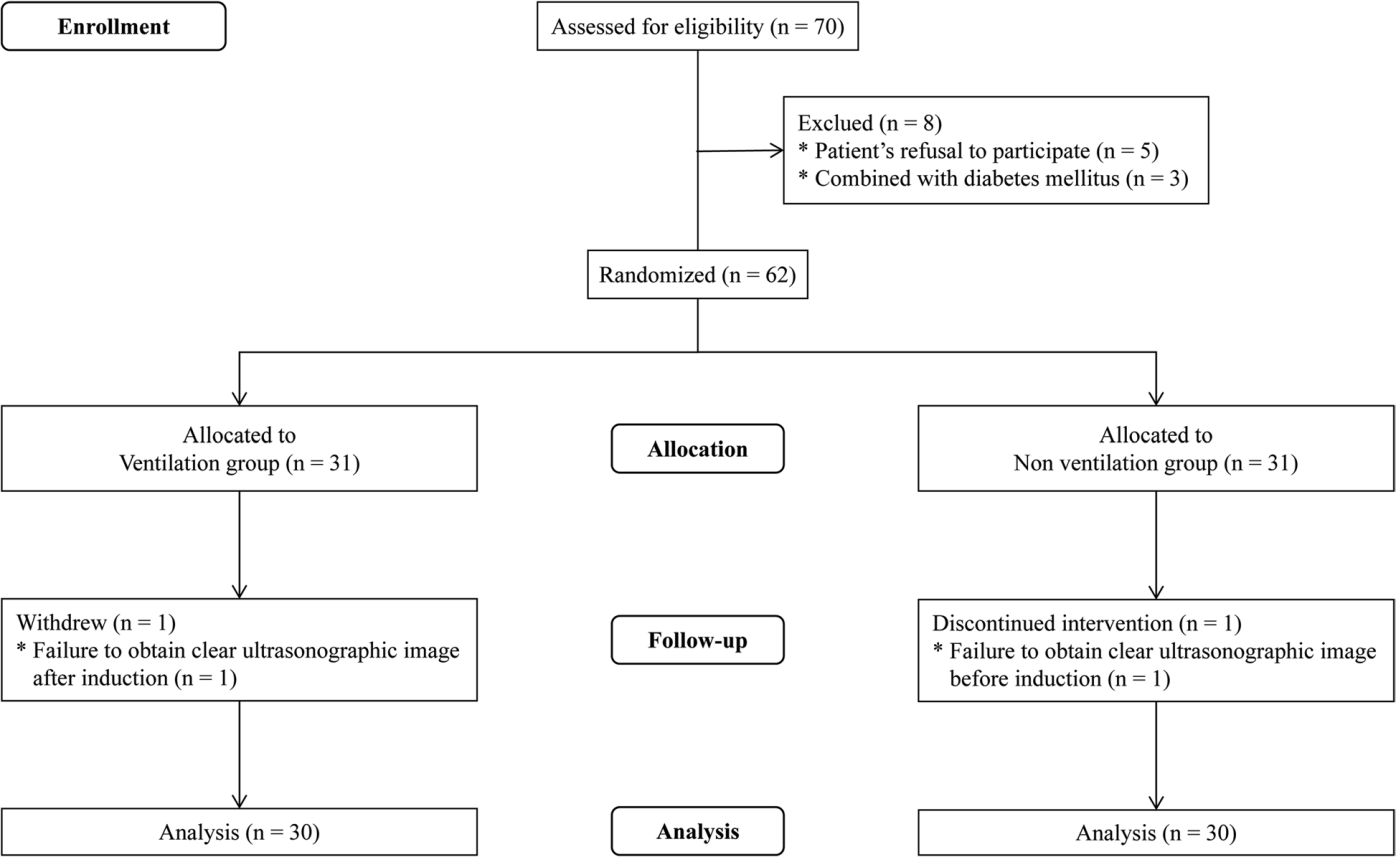
Flow diagram of the study according to consolidated standards of reporting trials

Seventy patients were evaluated for eligibility between December 2021 and May 2022. Five patients refused to participate, and three were excluded due to diabetes mellitus. The rest of 62 patients were randomized and allocated into ventilation group and non ventilation group respectively. Afterward, two patients withdrew; one in ventilation group due to failure to obtain a clear ultrasonographic image after induction, and one in non ventilation group due to failure to obtain a clear ultrasonographic image before induction. Finally, the analysis was based on the remaining 60 participants, 30 per group (Fig. 2). No data were missing for the patients included in the analyses. No adverse events occurred relevant to the study protocol, including hypoxemia (SpO_2_ < 90%), difficult facemask ventilation, gastric contents regurgitation, or aspiration. The first attempt at endotracheal intubation was successful in all patients.

There are no difference in the baseline characteristics and the induction data. The durations of loss of consciousness, endotracheal intubation, and induction were similar between the two groups (Table 1).

**Table 1:** Baseline characteristics and induction data of the participants

	Ventilation group (*n* = 30)	Non ventilation group (*n* = 30)
Male/Female [cases (%)]	17 (57) / 13 (43)	10 (33) /20 (67)
ASA status I/II [cases (%)]	22 (73)/8 (27)	24 (80)/6 (20)
Age (Years)	47 ± 11	43 ± 10
Height (cm)	162 ± 8	165 ± 7
Weight (kg)	59 ± 11	64 ± 8
BMI (kg m^−2^)	22 ± 3	24 ± 3
Fasting time (h)	13.0 [11.0 to 15.3]	13.0 [10.0 to 16.0]
Duration of losing consciousness(s)	60.0 [57.3 to 90.0]	60.0 [57.8 to 87.3]
Duration of endotracheal intubation (s)	51.5 [47.8 to 62.5]	54.5 [49.3 to 64.3]
Duration of anaesthesia induction(s)	361.0 [345.0 to 383.0]	362.5 [348.8 to 382.8]

Data are expressed as absolute number, *n* (%), mean ± SD and median [IQR] for each group, as appropriate

*ASA* American Society of Anaesthesiologists, *BMI* Body mass index

The median change of gastric antral cross sectional area in ventilation group was significantly higher than in non ventilation group (0.83 [− 0.34 to 3.79] vs. 0.10 [− 1.44 to 1.12], *P* = 0.001). The median difference was 0.64 cm^2^ (95% confidence interval [CI], 0.24 to 1.11). The gastric antral CSA before induction was similar between the two groups. After induction, the gastric antral CSA in non ventilation group was significantly lower than in ventilation group (Table 2).

**Table 2:** Comparison of gastric antral CSA before and after induction between two groups

Parameters	Ventilation group (*n* = 30)	Non ventilation group (*n* = 30)	*P*
Gastric antral CSA before induction (cm^2^)	3.4 [2.5 to 5.5]	3.6 [2.9 to 4.5]	1.00
Gastric antral CSA after induction (cm^2^)	4.6 [3.4 to 5.9]	4.0 [2.8 to 4.6]	0.02
The increase of gastric antral CSA (cm^2^)	0.83 [0.20 to 1.54]	0.10 [−0.11 to 0.56]	0.001

Data are expressed as median [IQR]

*CSA* Cross sectional area

**Fig. 3:**
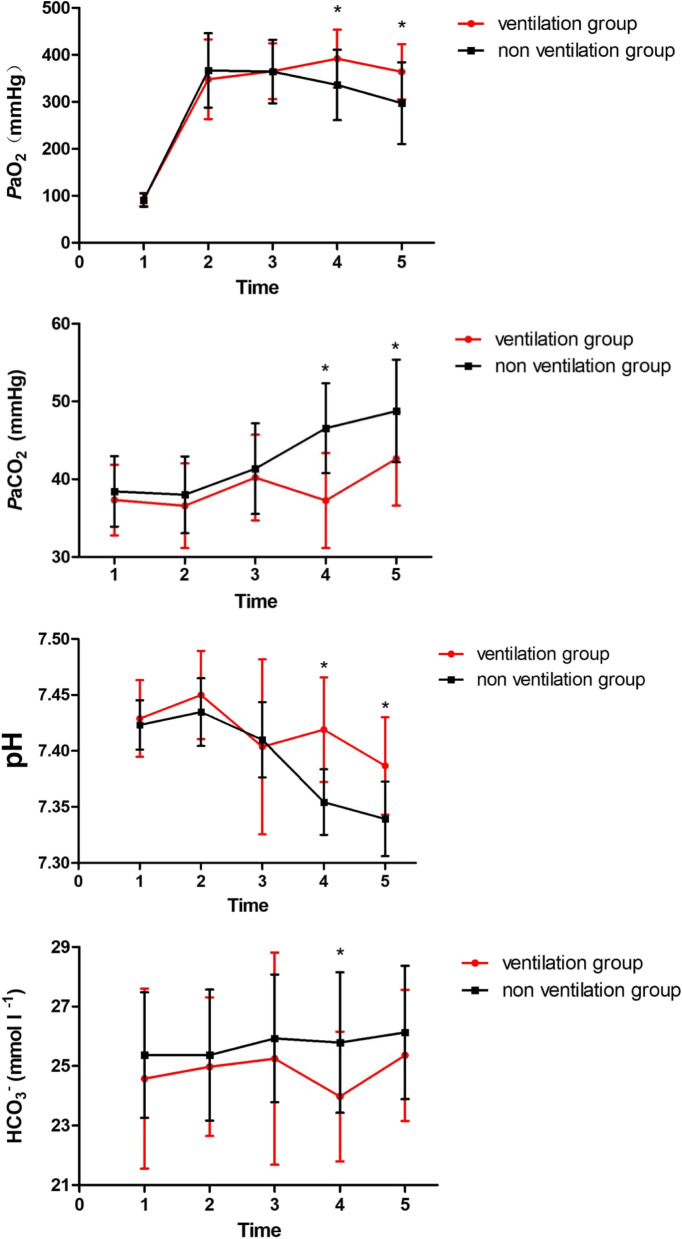
Change in *P*aO_2_, *P*aCO_2,_ pH and HCO_3_^−^ levels at different time points during anesthesia induction. *P < 0.01 between two groups. T1, baseline; T2, after preoxygenation; T3, when patient lost consciousness; T4, before endotracheal intubation; T5, after successful endotracheal intubation

After preoxygenation, the *P*aO_2_ increased in both groups. *P*aCO_2_ and HCO_3_^−^ levels reached their highest values at T5 in both groups. A two-way repeated- measures ANOVA with the Bonferroni post hoc-test and correction for *P* value was used to compare the PaO_2_, PaCO_2_, pH and HCO_3_^−^ at different time points between the two groups. At T4 and T5, the *P*aO_2_ in ventilation group was significantly higher than in non ventilation group (T4: 391.83 ± 61.53 vs. 336.23 ± 74.99 mmHg, F(1,29) = 10.68, *P* < 0.01; T5: 364.00 ± 58.65 vs. 297.13 ± 86.95 mmHg, F(1,29) = 15.14, *P* < 0.01), while the *P*aCO_2_ in non ventilation group was significantly higher (T4: 46.57 ± 5.78 vs. 37.27 ± 6.10 mmHg, F(1,29) = 36.03, *P* < 0.01; T5: 48.77 ± 6.59 vs. 42.63 ± 6.03 mmHg, F(1,29) = 17.58, *P* < 0.01) and the pH value in non ventilation group was significantly lower (T4: 7.35 ± 0.029 vs 7.42 ± 0.047, F(1,29) = 49.47, *P* < 0.01; T5: 7.34 ± 0.033 vs 7.39 ± 0.044, F(1,29) = 25.32, *P* < 0.01). At T4, the HCO_3_^−^ in non ventilation group was significantly higher (25.79 ± 2.36 vs. 23.98 ± 2.18 mmol l^− 1^, F(1,29) = 8.54, *P* < 0.01). Figure 3 depicts the changes in *P*aO_2_, *P*aCO_2_,  pH and HCO_3_^−^ throughout the different time points in both groups.

Eight patients (27%) in ventilation group and one (3%) in non ventilation group were defined as gastric insufflation under laparoscopy by the same surgeon (odds ratio, 10.5; 95% CI, 1.2 to 90.7; *P* = 0.03).

## Discussion

Laparoscopic cholecystectomy and partial hepatectomy are technically sensitive to gastric volume; therefore, the prevention of gastric insufflation is critically important. In the current study, we found that facemask oxygenation without ventilation had a milder effect on gastric volume than pressure-controlled ventilation with a targeted inspiratory pressure of 20 cmH_2_O. Using this technique during the anesthesia induction, no adverse events occurred.

General anesthesia impairs the esophageal sphincter tension and protective reflexes of upper respiratory tract, thus increasing the risk of gastric contents regurgitation and aspiration [14, 15]. Perioperative aspiration can result in severe complications, such as aspiration pneumonia, whose mortality rate was as high as 5% [16]. According to previous studies, 2.7–6.2% of fasting patients were defined as a sonographic full stomach state before elective surgery [17, 18]. Furthermore, despite following the fasting guidelines, 13% of the patients planned for elective laparoscopic cholecystectomy for symptomatic gallbladder disease appeared as a full stomach, determined by ultrasound imaging, before the anesthesia induction [19]. Continuous positive airway pressure using a facemask can result in gastric insufflation. The excessive presence of air in the stomach can cause a raise in gastric pressure, leading to gastric content regurgitation and possibly hemodynamic and pulmonary failure [20].

During anesthesia induction, hand-controlled, pressure-controlled, and volume-controlled facemask ventilation are the assisted ventilation modalities typically used. Pressure-controlled ventilation provides the same tidal volume as volume- or hand-controlled ventilation, with a lower inspiratory pressure, inspiratory peak velocity, and airway peak pressure, and the incidence of gastric insufflation is reduced.^5-7^ Therefore, facemask pressure-controlled ventilation was chosen in our study. To date, the optimal inspiratory pressure for pressure-controlled facemask ventilation during anesthesia induction remains to be determined. Previous studies suggest that the peak inspiratory pressure should be < 20 cmH_2_O. It is reported that an inspiratory pressure of 15 cmH_2_O resulted in a lower occurrence of gastric insufflation, while providing an acceptable tidal volume [21]. However, gastric insufflation could not be completely avoided, even with an inspiratory pressure of 10 cmH_2_O [21]. Theoretically, if facemask oxygenation without ventilation is adopted during apnea in the induction of general anesthesia, gastric insufflation and increase in intragastric pressure could be avoided, as suggested by the results of our study.

The traditional remedy for gastric insufflation caused by facemask ventilation with positive pressure is to release the gas using a nasogastric tube, for laparoscopic cholecystectomy or partial hepatectomy. However, it is challenging and traumatic to place a nasogastric tube in an anesthetised patient. Preoperative insertion of a nasogastric tube could prevent gastric insufflation during induction, though it might also damage the nasopharyngeal mucosa and result in erosion and bleeding. Moreover, it may cause tears, nausea, vomiting, cough, and other symptoms, causing discomfort to the patients. Therefore, the adoption of facemask oxygenation without ventilation during apnea in induction in patients undergoing laparoscopic cholecystectomy or partial hepatectomy is beneficial to avoid nasogastric tubes and reduce unnecessary damage.

In ventilation group, eight patients had marked gastric insufflation, which affected the surgical field of vision and required a nasogastric tube for gas release. This finding may be related to the large tidal volume of positive-pressure facemask ventilation and the absence of an oropharyngeal tube. In addition, during anesthesia induction, the use of propofol and remifentanil leads to a pressure decrease in the upper and lower esophageal sphincters. Furthermore, we employed rocuronium during anesthesia induction. As a non-depolarizing muscle relaxant, rocuronium can decrease the tone of the upper esophageal sphincter [22], thereby increasing the incidence of gastric insufflation. One patient in non ventilation group also experienced gastric insufflation. We speculate that active swallowing of oxygen during preoxygenation was the main reason for this occurrence.

Anesthesia induction usually inhibits or eliminates spontaneous breathing, and the prolonged absence of ventilation can lead to hypoxemia and hypercapnia. Tolerable apnea time or safe apneic time is defined as the period with SpO_2_ maintained > 90%, and it can be extended by adequate preoxygenation. Arterial blood gas analysis was used to assess the ventilation during induction. In both groups, the *P*aO_2_ was obviously increased after preoxygenation until endotracheal intubation was established, suggesting the important role of preoxygenation in enhancing the oxygen reserve. It can delay the desaturation during apnea in the non ventilation group, reducing the difference of *P*aO_2_ between the two groups at T4 and T5. Before and after endotracheal intubation, the *P*aO_2_ in non ventilation group was significantly lower than in ventilation group, not only because of continuous oxygen consumption but also impaired oxygen exchange. Preoxygenation with pure oxygen can lead to atelectasis [23]. Anesthesia induction with a muscle relaxant can cause the loss of respiratory muscle tone, resulting in a fall in the resting lung volume and functional residual capacity [24]. This fall facilitate airway closure and gas absorption, finally causing alveolar collapse, increasing the area of atelectasis [25]. Blood perfusion of the atelectasis can lead to shunt while airway closure generates areas with low ventilation/perfusion ratios, both of which impaired oxygenation. Furthermore, the residual nitrogen in the alveoli combined with the accumulation of carbon dioxide reduces the pressure gradient of oxygen, weakening the effect of apneic oxygenation and accelerating the reduction in *P*aO_2_ [26].

During apnea, the accumulation of CO_2_ leads to respiratory acidosis, resulting in a compensatory gradual increase in plasma HCO_3_^−^ levels and hypercapnia. In this study, the *P*aCO_2_ before and after endotracheal intubation in non ventilation group were significantly higher than in ventilation group while the pH value was significantly lower. On the one hand, the elimination of CO_2_ is impeded during apnea. On the other hand, pulmonary vascular shunt caused by atelectasis leads mixed venous blood with higher CO_2_ content to flow directly to the arterial side [27], exacerbating accumulation. However, transient respiratory acidosis and hypercapnia due to the accumulation of CO_2_ is permitted, even rapid hypercapnia does not result in significant hemodynamic disturbances [28–30]. Futhermore, it can be quickly redressed by mechanical ventilation after endotracheal intubation.

Using transesophageal echocardiography, a clearer gastric ultrasonographic image can be obtained [31]; however, this modality is invasive and requires additional equipment. In contrast to other traditional methods like gastric impedance monitoring, scintigraphy and paracetamol absorption tests, the bedside transabdominal ultrasonography used in our study is a straightforward and non-invasive technique to obtain the image of gastric antrum and evaluate the gastric content [32], helping the anesthesiologists to make decisions. Nevertheless, in clinical practice, the significance of the present threshold for a full stomach determined by gastric ultrasonography is still unclear [33]. The reported incidence of a sonographic full stomach (2.7–6.2%) [17, 18] is markedly above the clinical incidence of pulmonary aspiration (0.031%) [16]. Currently, the significance of a sonographic full stomach on the risk of pulmonary aspiration is indefinite because no compelling evidence emerged from randomized controlled trials.

Our study has several limitations. First, it was performed in a single center with a relatively small sample size. Second, the two-handed mask-hold technique conduced to avoiding air releases from the mask, possibly increasing the incidence of gastric insufflation. Third, the ultrasonographic measurements of the gastric CSA were performed by a single operator. It would have been preferable to have two physicians performing them to assess the inter-operator variability. Fourth, arterial blood gas analysis was not performed after a period of ventilation to analyze the changes in relevant parameters. Fifth, using facemask oxygenation without ventilation could be risky for patients with difficult endotracheal intubation, though there were no patients with hypoxemia in our study. Finally, this study included only healthy adult non-obese patients with normal cardiopulmonary function; thus, the efficacy and safety of facemask oxygenation without ventilation remain to be determined in the elderly or in patients with obesity, cardiac insufficiency, or lung disease.

In conclusion, facemask oxygenation without ventilation during apnea in the induction of general anesthesia has a milder effect on gastric volume than facemask pressure-controlled ventilation with a targeted inspiratory pressure of 20 cmH_2_O, without adverse events. This technique could be useful for laparoscopic cholecystectomy or partial hepatectomy. As this result was derived from healthy non obese adults, subsequent validation studies for the elderly or patients with obesity, cardiac insufficiency, or lung disease are required.

## Data Availability

The datasets used and analysed during the current study are available from the corresponding author on reasonable request.

## Abbreviations

CSA: Cross sectional area
*P*aO_2_: Arterial partial pressure of oxygen
*P*aCO_2_: Partial pressure of carbon dioxide
HCO_3_^−^: Bicarbonate
SpO_2_: Oxygen saturation
ASA: American Society of Anesthesiologists
BMI: Body mass index
SD: Standard deviation
IQR: Inter quartile range
